# The Role of the Slc39a Family of Zinc Transporters in Zinc Homeostasis in Skin

**DOI:** 10.3390/nu10020219

**Published:** 2018-02-16

**Authors:** Bum-Ho Bin, Shintaro Hojyo, Juyeon Seo, Takafumi Hara, Teruhisa Takagishi, Kenji Mishima, Toshiyuki Fukada

**Affiliations:** 1 Division of Pathology, Department of Oral Diagnostic Sciences, School of Dentistry, Showa University, Tokyo 142-8555, Japan; bbh82429@gmail.com (B.-H.B); mishima-k@dent.showa-u.ac.jp (K.M.); 2 Osteoimmunology, Deutsches Rheuma-Forschungszentrum, 10117 Berlin, Germany; Shintaro.Hojyo@drfz.de; 3 AmorePacific Corporation R&D Center, Yongin, Gyeonggi-do 446-729, Korea; yeon8687@amorepacific.com; 4 Faculty of Pharmaceutical Sciences, Tokushima Bunri University, Tokushima 770-8055, Japan; t-hara@ph.bunri-u.ac.jp (T.H.); t.takagishi@ph.bunri-u.ac.jp (T.T.); 5 RIKEN Center for Integrative Medical Sciences, Yokohama 230-0042, Japan

**Keywords:** zinc, skin, homeostasis, transporter

## Abstract

The first manifestations that appear under zinc deficiency are skin defects such as dermatitis, alopecia, acne, eczema, dry, and scaling skin. Several genetic disorders including acrodermatitis enteropathica (also known as Danbolt-Closs syndrome) and Brandt’s syndrome are highly related to zinc deficiency. However, the zinc-related molecular mechanisms underlying normal skin development and homeostasis, as well as the mechanism by which disturbed zinc homeostasis causes such skin disorders, are unknown. Recent genomic approaches have revealed the physiological importance of zinc transporters in skin formation and clarified their functional impairment in cutaneous pathogenesis. In this review, we provide an overview of the relationships between zinc deficiency and skin disorders, focusing on the roles of zinc transporters in the skin. We also discuss therapeutic outlooks and advantages of controlling zinc levels via zinc transporters to prevent cutaneous disorganization.

## 1. Introduction

Zinc is an essential micronutrient obtained from food and drink [1]. Zinc is abundantly contained in mollusks such as oysters and in crustaceans such as crabs and crayfish [2]. The biological role of zinc was first revealed in 1869 by Raulin, who showed that zinc is essential for the growth of *Aspergillus niger* [1]. Subsequently, its importance for rodent growth was demonstrated using experimental rats in 1934 [1,3]. However, zinc’s physiological role in humans was unknown until 1961 when zinc deficiency in humans was discovered [4]. Zinc is associated with growth, gonad development, immune function and pregnancy outcome improvement, and hair loss prevention [1,5,6]. In 1961, growth retardation and hypogonadism observed in Iranian and Egyptian adults were reported to be associated with zinc deficiency, which was closely related to dietary habits [4,7,8]. Middle Eastern diets typically include bread and beans, which contain large quantities of phytate [9]. Phytate has a negative effect on zinc absorption, thereby causing zinc deficiency. Zinc deficiency is also observed in vegetarians, single people, alcoholics, dieters, pregnant women, and the malnourished in developing and developed countries [4]. In this review, we outline the characteristics of zinc transporters and identify skin phenotypes of their knockout mouse models and human skin genetic diseases caused by mutations in zinc transporters. Furthermore, we discuss how zinc deficiency impacts normal skin development and homeostasis, based on the most recent research.

## 2. Zinc Homeostasis in Skin

### 2.1. Skin Structure

The skin largely consists of three layers [10,11]. The epidermis functions as a barrier to protect the interior from direct contact with the external environment. The dermis supports the epidermis by filling the skin volume with fibers. The hypodermis is present under the dermis and is composed of subcutaneous fat layers [10,11,12]. 

The epidermis is a cell layer composed of keratinocytes, the basal layer of which contains progenitor cells (called basal cells) at the interface with the dermal layer. These cells gradually proliferate perpendicularly to the basal layer. At the same time, they differentiate into spinous cells and induce keratinization while undergoing enucleation [10]. Spinous cells are first differentiated from the basal layer and produce keratin, which contributes to tight cell-to-cell adhesion [10,11]. These cells then differentiate into granular cells that are rich in keratohyalin and eject lipids and proteins, as well as connect keratin fibers with higher density [10,11,12]. Next, the granular cells immediately die after denucleation and form corneocytes that are eventually pushed up to the surface of the skin, resulting in a firm stratum corneum, the outermost layer of the dermal barrier.

Most of the dermis consists of collagen, elastin, and the polysaccharide hyaluronan, which is produced by fibroblasts [10,11,12]. There are various nerves, blood vessels, hair follicles, sweat glands, macrophages, and T-cells, which play important roles in the secondary function of skin sensation and immunity [4,13,14,15]. Upon aging or ultraviolet (UV) stimulation, the levels of these substances are decreased, leading to the production of matrix metalloproteinases (MMPs), which are degrading enzymes that reduce skin volume.

The hypodermis is a subcutaneous organization of adipocyte-derived lipids [4,13,14,15]. Fat tissue is important for maintaining body temperature in humans. Unlike reptiles, which change temperature depending on the environment, humans, with a thin epidermis, develop fat tissue in the hypodermis to maintain body temperature and protect the body organs. Therefore, surplus energy can be stored in the hypodermis, and nerves and blood vessels larger than the dermis are safely preserved from external impact, completing the complex human body as an organic and safe system.

### 2.2. Zinc Transporters

**Figure 1:**
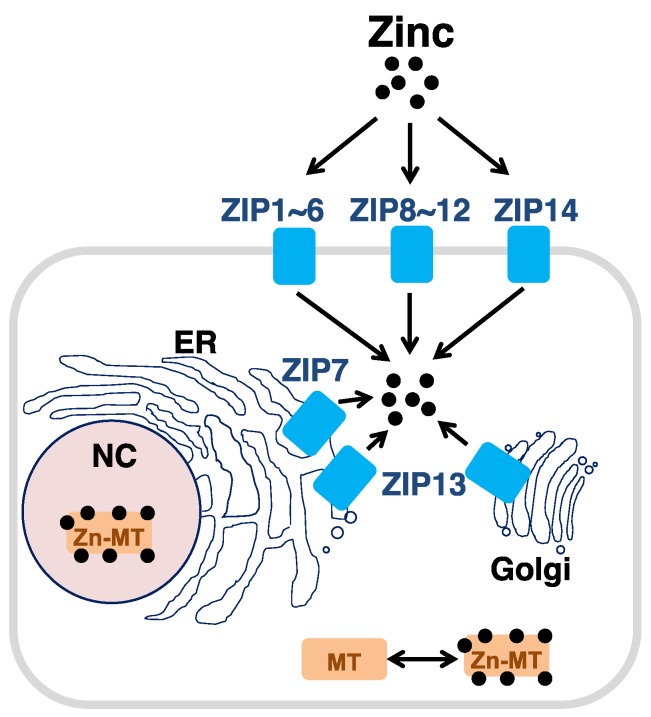
Zinc transporters and metallothionein (MT) are involved in intracellular zinc homeostasis. NC; nucleus, ER; endoplasmic reticulum, Zn; zinc.

Intracellular zinc homeostasis is tightly regulated by zinc transporters and metal binding proteins, known as metallothioneins (MTs) (Figure 1) [16]. Because zinc is a metal ion that cannot pass through the cell wall, where lipid is abundant, cells must use carriers to maintain intracellular zinc homeostasis [16,17]. There are two types of zinc transporters: ZIP, which transports zinc into cells from extracellular regions or the luminal side of intracellular compartments and is dependent on zinc concentration, and ZnT, which transports zinc to the exterior of cells or the lumen side from cell cytoplasm. There are 14 ZIP and 10 ZnT family members in humans, and their expression patterns and intracellular locations vary depending on the cell type, developmental stage, and Zn status [18]. Currently, the structure of the ZIP family has not been clarified, but it is thought to possess a domain that penetrates the cell membranes approximately eight times and constitutes a homodimer or a heterodimer with other ZIP members [19,20]. Both ends of ZIP family member peptides face the extracellular or luminal side, with a variety of N-terminal domains, while the intracellular domain has two lengths [19]. In particular, the two domains contain a large number of histidine residues that can bind zinc. Proteoliposome studies using bacterial homologs have suggested that ZIP family members have transport mechanisms such as channels that are independent of other ionic concentrations or adenosine triphosphate (ATP) [21]. In some cases, the filter allowing the passage of zinc is thought to flexibly transport other metals with physicochemical properties similar to those of zinc such as cadmium. For instance, ZIP14 is responsible for transporting iron and manganese in the liver [22,23,24]. ZIP8 and ZIP14 are symporters that carry metal and biscarbonate ions [25].

ZIP4 is an intensively studied transporter expressed in enterocytes of the small intestine and plays a first gate role in absorbing zinc into the body [26,27]. When zinc is deficient, the N-terminus of ZIP4 is cleaved and the remaining truncated protein migrates to the cell surface to help absorb zinc [28,29,30]. *ZIP4* is a causative gene for acrodermatitis enteropathica (AE), in which systemic zinc deficiency occurs because of impaired intestinal zinc transport [31]. Dozens of pathogenic mutations have been identified. Recent studies showed that single-nucleotide polymorphisms (SNPs) in *ZIP4* differ significantly among various regions of sub-Saharan Africa, where people exhibit high sensitivity to zinc deficiency [32]. Some people have a leucine-to-valine (Leu372Val) replacement in the ZIP4 protein that allows for the expression of small amounts of ZIP4 on cell surfaces and thus transport a low amount of zinc, whereas others show the pathogenic mutations Leu372Pro and Leu372Arg, which prevent ZIP4 from migrating to the cell surface. Homozygous knockout of *ZIP4* in an AE mouse model shows embryonic lethality [27], indicating that ZIP4 is essential for embryonic development in mice. A conditional *Zip4*-knockout mouse in which a *Zip4* allele is specifically deleted by Cre-Lox recombination controlled under the villin promoter has also been generated [33]. These mice show collapse of the stem cell niche and integrity of the small intestine, but do not present any skin abnormality.

ZIP10 is mainly localized to the cell surface and carries zinc in B-cells [34,35]. A conditional *Zip10*-knockout mouse in which a *Zip10* allele is specifically deleted by Cre-Lox recombination controlled under the keratin 14 promoter shows impaired skin barrier [36]. ZIP7 and ZIP13 are present in the endoplasmic reticulum (ER)/Golgi and play important roles in zinc homeostasis within these compartments [37,38]. A conditional *Zip7*-knockout mouse under the collagen 1 promoter results in a thin epidermis; thus, both ZIP7 and ZIP10 are essential for skin homeostasis [39]. In contrast to ZIPs, ZnTs are unique secondary transporters with Y-type and transport zinc using a concentration gradient of a partner transport substrate such as hydrogen ions [40,41]. As in the ZIP family, the ZnT group is present on the cell surface or within the cells depending on the cell type, and thus plays an indispensable role in zinc export [17].

As ZIPs and ZnTs, several transporters and channels were also found to mediate zinc influx. Although their main substrates do not appear to be zinc, transient receptor potential (TRP) channels, divalent metal transporter (DMT1), *N*-methyl-d-aspartate receptors (NMDA), amino-3-hydroxy-5-methyl-4-isoxazolepropionate receptors (AMPA-Rs), and voltage-dependent calcium channels (VDCCs), are reported to transport zinc [42,43]. However, their involvement in skin development and homeostasis is unknown. 

### 2.3. MTs

MTs were first purified from the cortex of horse kidneys in 1957 [44]. MTs are small proteins (molecular weight less than 7 kDa) containing more than 33% cysteine residues and facilitate the storage of zinc, cadmium, and copper, etc. with high thermal stability [45,46]. MTs are important for acquiring resistance to epithelial apoptosis mediated by reactive oxygen species, possibly through their antioxidant activities [15,47]. Currently, more than 10 isoforms have been identified in humans, located in both the cytoplasm and nucleus [48,49,50]. Mice lacking *Mt1* or *Mt2* exhibit no specific skin abnormalities [51]. However, the epidermal swelling caused by cholera toxin or UV-B irradiation is not observed in *Mt1/2*-double knockout mice [52]. In a mouse wound healing model, zinc enrichment in hair follicles was found in parallel with increased MT1 and MT2 expression during the wound healing process [53], suggesting that MT1 and MT2 play important roles in epidermal proliferation in certain situations. 

### 2.4. Zinc Levels in Skin

The expression of MTs is induced by metal-responsive transcription factor 1 (MTF1) in a zinc concentration-dependent manner. Therefore, many studies have indirectly monitored the quantity of zinc in cells and tissues by measuring MT expression levels [19,54,55]. MT in the skin is mainly accumulated in progenitor cells and initial spinous cells at the bottom of the epidermis near the basal layers [56]. MTs are also found in out-root sheath cells of hair follicles and epidermal stem cells [57], which are undifferentiated cells with common features and show strong proliferative capacity when necessary [11,12]. Given that zinc is required as a structural component or activating cofactor for over 300 enzymes and other proteins related to cell proliferation, survival, and differentiation, undifferentiated cells with proliferative capacity contain high amounts of zinc [17,46]. It is known that zinc has excellent efficacy for treating and regenerating skin wounds. Indeed, zinc deficiency causes delayed growth with skin anomalies [1,58]. Therefore, zinc is crucial for epidermal stem cells that regenerate inter-follicular epidermis, sebaceous gland, and hair follicle cells by rapidly migrating and dividing basal cells in the early stages of differentiation and scar tissue formation. Treatment with materials that thicken the epidermis have been shown to increase the amount of MT1 in such regions [52,57]. Fibroblasts are sparse in the dermis and do not undergo rapid cell division, suggesting that the dermis contains less zinc than the epidermis. In fact, zinc is present at 60 µg/g in the epidermis and 40 µg/g in the dermis [13,59]. Furthermore, it has been reported that the upper dermis contains higher zinc levels than the lower dermis [59]. This difference may be attributed, in part, to mast cells that contain a large quantity of zinc in the granules. Recently, the skin zinc concentration was measured by synchrotron radiation high energy X-ray fluorescence [60]. This study suggested that the greatest amount of dermal zinc exists in the stratum spinosum, a finding that somewhat differs from those of previous studies that showed a large quantity of zinc in spinous and granular cells compared to basal cells. Given that it is difficult to accurately measure the concentration of specific metals without interference from other metals, a new method for precise measurement of zinc concentration should be developed.

### 2.5. Zinc Deficiency in Skin

There are two major causes of zinc deficiency in humans. First, zinc deficiency is caused by a diet that is low in zinc content. Approximately 17% of the world’s population is confronting health risks related to zinc deficiency, particularly in developing countries. However, in even developed countries, vegetarians, pregnant women, and singles also suffer from zinc deficiency. Second, zinc deficiency can be caused by genetic defects. In 1988, it was reported that babies with severe skin diseases and hair loss were diagnosed with transient neonatal zinc deficiency [61], but recovered by eating normal infant foods [62]. Their mothers have a variety of mutations in alleles of the zinc transporter ZnT2 [63].

When ZnT2 function is impaired, mammary epithelial cells do not release zinc into breast tissue [63]. Therefore, patients with transient neonatal zinc deficiency cannot expect to increase zinc concentrations in the breast milk unless zinc is prescribed. Mice carrying mutations involving ZnT4 exhibit lethal milk (*lm*) syndrome, causing dermatitis and alopecia, but this is irrelevant to humans. Thus, clinical features according to the zinc transport species involved differ among species [64]. Although zinc deficiency is diagnosed by direct measurement of zinc concentrations in the blood, accuracy can be low and perturbed even under healthy conditions. Therefore, the development of new zinc biomarkers has been widely pursued for more precise measurement of zinc levels (e.g., erythrocyte linoleic acid:dihomo-y-linolenic acid (LA:DGLA) [65]. 

Zinc deficiency initially causes skin problems such as dermatitis, alopecia (thin and sparse hair), acne, eczema, dry, scaling skin, delayed wound healing, and oral ulceration, as well as problems such as stomatitis. [1,17,58,66]. Dermatitis due to zinc deficiency is termed as AE. Brandt’s syndrome also includes autosomal recessive metabolic disorders associated with AE, which is accompanied by inherited genetic abnormalities with low zinc distribution. In AE patients, personality disorders are observed, with blistering of the skin (dry skin), hair loss from the scalp, eyebrows, and eyelashes, glossitis, and pustules. Therefore, secondary infections can be easily caused by pathogens such as *Staphylococcus aureus* and fungi such as *Candida albicans* in the weakened epidermis due to zinc deficiency. Recent reports revealed that keratinocytes produce excess amounts of ATP, which induces inflammation upon contact with irritants in individuals with zinc-deficient skin [67]. In zinc-deficient mice, Langerhans cells (LCs), which can neutralize harmful ATP by surface CD39, are greatly reduced in the skin, resulting in augmented skin inflammation by ATP. In AE patients, the number of LCs is also decreased, and is recovered by zinc treatment. The effects of zinc deficiency have also been studied on a cellular basis. When keratinocytes in monolayer culture are scratched, zinc is released from these cells [68]. Subsequently, zinc binds to zinc-sensing receptors and induces cell proliferation in an autocrine/paracrine manner, suggesting that zinc is required for skin regeneration. In fact, the removal of zinc by *N*,*N*,*N*,*N* tetrakis (2-pyridylmethyl) ethylenediamine (TPEN) during keratinocyte culture induces caspase-3 activity and DNA fragmentation followed by apoptosis [69]. Zinc also has an anti-inflammatory effect and reduces tumor necrosis factor-α production by keratinocytes [59]. A recent study demonstrated that R1 and EVER2 proteins found in epidermodysplasia verruciformis (EV) patients with high susceptibility to papillomaviruses causing skin cancer interact with ZnT1 to control the zinc balance in keratinocytes, suggesting the importance of maintaining intracellular zinc homeostasis in skin cancer induced by viral infection [70].

## 3. Zinc Transporters and Skin Disorders

### 3.1. Epidermis

**Figure 2:**
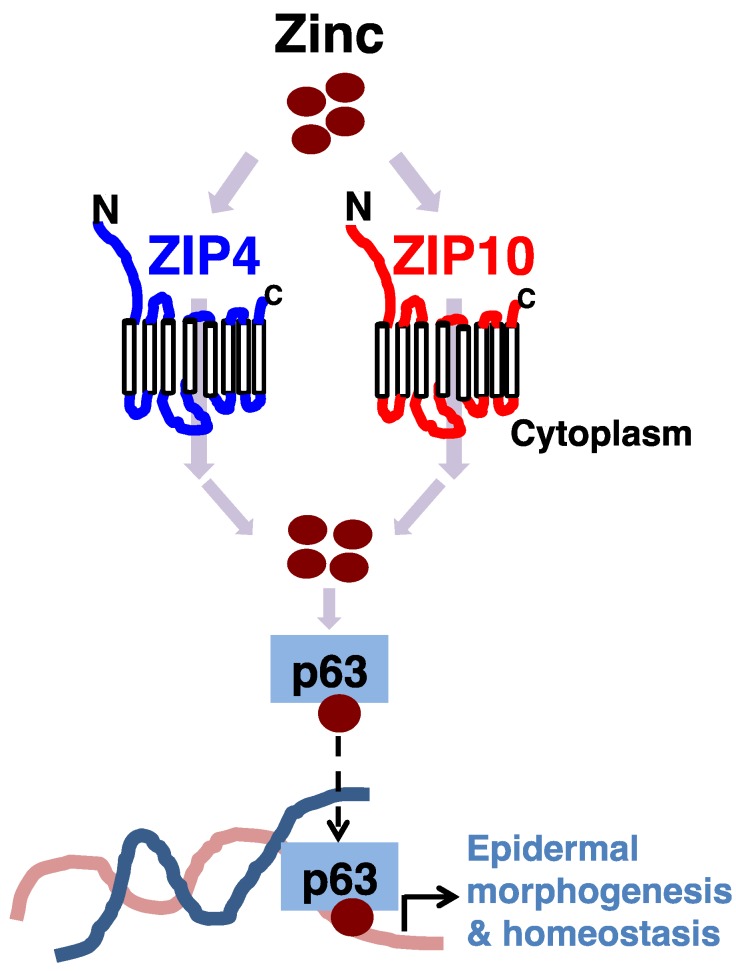
ZIP4 and ZIP10 support p63 activity for adult epidermal homeostasis. ZIP4 and ZIP10 supply zinc to epidermal master regulator p63 for their activity. Zinc deficiency leads to improper function of p63, resulting in epidermal hypoplasia. Zinc is depicted as a brown circle.

Zinc transporters known for their role in the epidermis are ZIP4 and ZIP10. *ZIP4* is actively expressed in human but not in mouse skin [33,71]. Recent skin equivalent experiments with human keratinocytes demonstrated the importance of ZIP4 in epidermis formation [56]. When *ZIP4* is knocked-down by siRNA, epidermal hypoplasia occurs, accompanied by decreased activity of p63, an master regulator with a zinc binding site that promotes the proliferation and differentiation of epidermal progenitor cells in epithelium formation (Figure 2) [56,72,73]. When zinc is deficient, nuclear translocation of p63 is disturbed, followed by abnormal epidermal formation (Figure 2) [56]. Detailed analysis revealed that the 205th Cys among the four Cys residues in the zinc binding site plays an important role in post-modification of p63, suggesting that zinc deficiency affects the DNA binding affinity of p63 involved in signal transduction. Since Zn affects the structure and functions of many proteins [17], ZIP4 may not only target the p63, but also influence the structure and activity of other zinc binding enzymes for epidermal growth. With regard to this issue, further studies are needed to clarify the relationship between ZIP4 and AE. Interestingly, it was recently found that natural products such as soybean extracts increase the expression of ZIP4 [74]. Therefore, this can be a starting point for controlling the rate of zinc absorption.

Of note, the murine and human epidermis have several different characteristics. Overall, the human epidermis is thicker than the mouse epidermis [11,12]. The stratum corneum shows a solid and thick-layered structure, and has a rete ridge structure because of the diversity of the number of spinous layers. The patterns of epidermal stem cells in the basal layer also differ between mouse and human. Therefore, the use of mouse models in human epidermal studies should be carefully assessed. 

**Figure 3:**
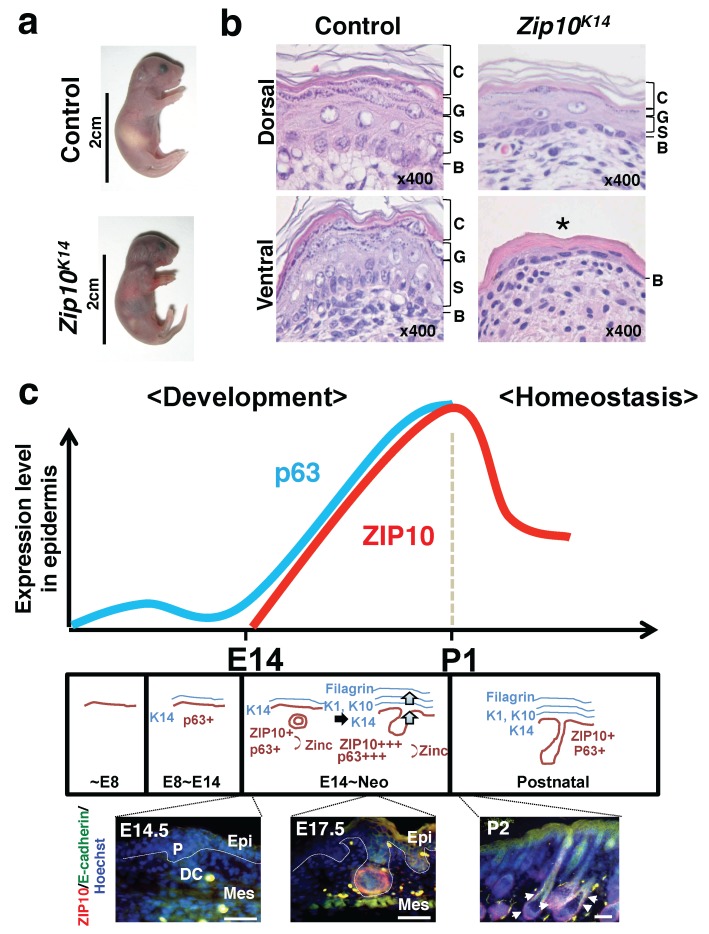
ZIP10 reinforces p63 function for epidermis development. (**a**) ZIP10 deficiency leads to epidermal hypoplasia in mice. (**b**) Hematoxylin and eosin staining revealed dorsal epidermal hypoplasia and ventral embryonic epidermis (asterisk) in P1 *Zip10^K14^* mice. C, cornified layer; G, granular layer; S, spinous layer; B, basal layer. (**c**) Model for ZIP10’s involvement in p63 function during epidermis development. ZIP10, whose expression is elevated from E14, contributes to the activities of increased p63 by supplying zinc. This allows p63 to properly bind to DNA for initiating gene expression for epidermis development, including cell proliferation and stratification. DC, dermal condensate; Epi, epithelium; Mes, mesenchyme; P, placode (Scale bar, 100 μm). Red (E17.5) and arrows (P2) indicate the ZIP10 protein expression. Modified from Bin et al. [36].

ZIP10 also plays important roles in epidermal development [36]. In situ hybridization analysis using mouse whole body sections showed that ZIP10 expression appears in hair follicles near embryonic day 14 (E14) during epidermal development, after which it gradually increases. In a postnatal phase after the completion of epidermis formation, ZIP10 expression is decreased. In the epidermis, ZIP10 accumulates in the outer root sheath region of the hair follicle, which is rich in various Lgr6-positive epidermal stem cells that migrate to the sebaceous glands, hair follicles, and wound space, when epidermis is regenerated upon injury. Consistent with this, next-generation sequencing analysis confirmed that *Zip10*-expressing cells co-express *Lgr6*. A conditional knockout mouse line in which *Zip10* was specifically deleted in Keratin14-expressing cells was born with severe epidermal hypoplasia (Figure 3a,b). In these mice, the p63-positive basal layer formed normally, and slight stratification of the dorsal part of the epidermis occurred by E14. However, such mice fail to form a thick and firm epidermis following rapid proliferation and differentiation of epidermal progenitor cells after E14 when the expression of both ZIP10 and p63 is increased in wild-type mice (Figure 3b,c). Further detailed analysis revealed that ZIP10 augments the activity of p63 by regulating its nuclear translocation (Figure 3c).

### 3.2. Dermis

**Figure 4:**
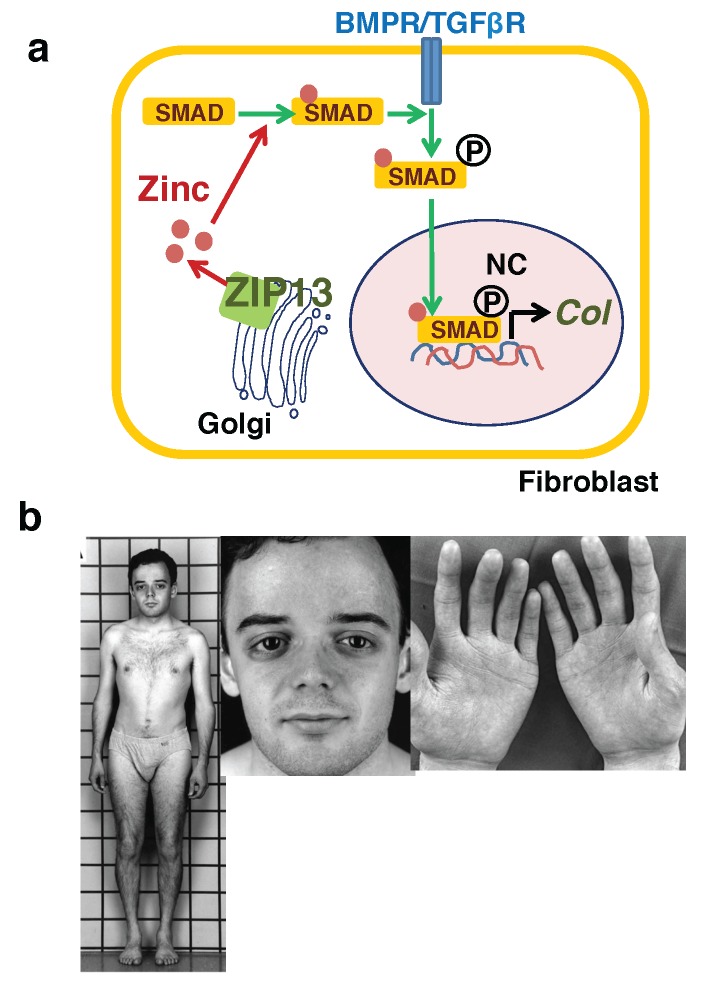
Ehlers–Danlos syndrome spondylodysplastic type 3 is caused by impaired collagen production due to ZIP13 dysfunction. (**a**) ZIP13 supplies zinc (brown circle) to SMAD proteins for their nuclear translocation. Phosphorylatoin is depicted as circled P. (**b**) The elder affected sib is shown at age 22 years. Patient exhibits short stature with mildly shortened trunk, antimongoloid eye slant with lack of periorbital tissue, thin and finely wrinkled skin on the palms of the hands. BMPR/TGFβR, BMP receptor/TGF-β receptor; *Col*, collagen. Modified from Fukada et al. [38].

Zinc transporters known to be important in the dermis are ZIP 7 and ZIP 13 [37,38]. Both ZIP7 and ZIP13 are present in skin cells and are mainly located in the ER and Golgi, respectively [19,37,38]. These two zinc transporters are closest to each other when a phylogenetic tree of ZIP family members is constructed. ZIP13 is also close to the zinc transporter of insects/bacteria [19]. ZIP13 is the smallest of the mammalian zinc transporters classified in the LIV-1 family, and thus has a shorter N-terminus and intracellular domain 2 than other transporters. The most abundant amino acid in ZIP7 is histidine, which is a characteristic of the LIV-1 family whose role is unknown; this is also the case for the methionine-rich motif that is largely conserved in copper transporters for copper binding and storage [19,75]. During functional analysis of zinc carriers, the first association of the zinc transporter with skin was found in *Zip13*-knockout mice [38]. The *Zip13*-knockout mice showed impaired collagen production with abnormal morphology of collagen-producing cells and shrunken cartilage with defective chondrocyte differentiation. The number of collagen fibers in *Zip13*-knockout mice was remarkably reduced as visualized under an electron microscope, and skin cracking and swelling phenomena were also remarkable because of severely decreased skin strength. This abnormal collagen production was reflected in the mouse face features with an enophthalmos-like appearance, eye blindness, and down-slanting palpebral fissures. The fibroblasts isolated from *Zip13*-knockout mice exhibited dysfunction in the BMP/TGF-β signaling pathway, which is essential for collagen formation. In *Zip13*-knockout mice, the nuclear translocation of SMAD transcription factors in the BMP/TGF-β signaling pathway was impaired, while remaining intact in terms of their phosphorylation status (Figure 4a). Examination of the zinc distribution in *Zip13*-deficient fibroblasts with an electron probe X-ray micro analyzer revealed that the nuclear zinc content was reduced, while that in the Golgi was increased [38,76].

The phenotypes of *Zip13*-knockout mice are similar to those of patients with spondylocheiro dysplastic form of Ehlers–Danlos syndrome (Ehlers–Danlos syndrome spondylodysplastic type 3, EDSSPD3), with an extremely thin layer of dermis and many wrinkles. EDSSPD3 patients have similar phenotypes such as sagging of the eyes, cracking of the skin, and prominent blood vessels (Figure 4b) [38,76,77]. To date, two mutated Gly64Asp and ΔPhe-Leu-Ala residues in ZIP13 protein have been identified in patients with EDSSPD3. Each of these mutations results in rapid clearance of ZIP13 protein due to ER-associated degradation via valprotein (VCP)/ubiquitination/proteasome machinery [78,79]. Thus, the compounds that increase the activity of ZIP13 or slow the degradation of ZIP13 are considered as therapeutic drugs that can increase collagen synthesis. Since ZIP13 is not only expressed in the Golgi, but also in putative zinc stores [80], another potential mechanism of EDSSPD3 development may be due to non-smooth zinc transport from zinc stores to the cytoplasm in the absence of ZIP13, resulting in low zinc levels in the cytoplasm. If this is the case, this condition also may induce ER stress, as the cytoplasm is a main zinc source for zinc homeostasis in the ER. 

**Figure 5:**
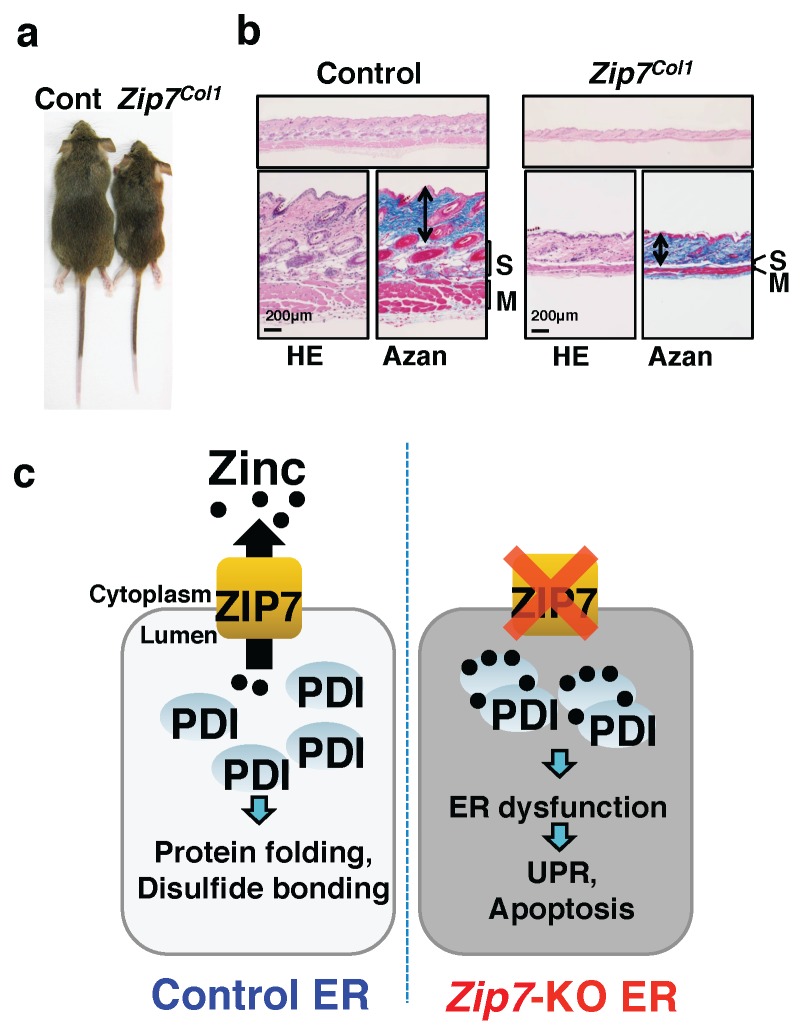
ZIP7 is essential for dermis formation. (**a**) Five-week-old female wild-type (WT) and *Zip7^Col1^* mice. (**b**) The thickness of the Azan-stained *Zip7^Col1^* dermis is reduced. M, muscle; S, Subcutis. (**c**) Models for the involvement of ZIP7 in ER function. When ZIP7 is dysregulated (right), the luminal zinc level is elevated, which would induce zinc-dependent aggregation of PDIs. Therefore, protein folding and disulfide bond formation would proceed aberrantly, leading to unfolded protein responses. ER, endoplasmic reticulum; KO, knockout; PDI, protein disulfide isomerase; UPR, unfolded protein response. Modified from Bin et al. [37].

ZIP7 is ubiquitously expressed in the ER of nearly all mammalian tissues [17,18] and maintains zinc homeostasis in this intracellular organelle, and the ER acts as a zinc reservoir, although a study showed that ZIP7 also exists in the Golgi [81]. *Zip13*-knockout mice display specific abnormalities in connective tissues irrespective of ZIP7 expression [37], suggesting that ZIP13 and ZIP7 play distinct physiological roles in connective tissue development. Similar to in *Zip13*-knockout mice, *Zip7*-conditional knockout mice, in which Cre-Lox recombination is under the control of the collagen promoter, dysfunction of connective tissues including the dermis and cartilage is observed (Figure 5a,b) [37,38]. The width of the growth plate in *Zip7*-knockout mice is reduced with a normal columnar structure, while that of *Zip13*-knockout mice is disorganized. Unlike *Zip13-*knockdown cells, *Zip7-*knockdown cells show elevated ER stress with increased zinc concentrations in the ER and protein aggregation of protein disulfide isomerase (PDI) [37]. In a *Zip7*-deficient environment, PDI does not function properly because of persistent sticking of zinc on the protein. In fact, *Zip7-*deficient cells fail to achieve the classical protein folding function in the ER, leading to ER stress, unfolded protein response, cell growth inhibition, and apoptosis (Figure 5c). Taken together, this series of studies indicate that proper intracellular zinc distributions in the ER and Golgi by ZIP7 and ZIP13, respectively, are critical for collagen synthesis.

### 3.3. Hypodermis

*Zip7-* and *Zip13*-knockout mice display reduced thickness of subcutaneous fat and dermal layers [37,38]. In EDSSPD3 patients, the subcutaneous fat layer is also significantly reduced in the aging skin [38]. Adipocytes play a major role in the formation of subcutaneous fat layers, and fibroblasts produce collagen for dermal formation. Thus, the abnormality of fat layers in *Zip7-* and *Zip13*-knockout mice may be attributed to impaired differentiation of mesenchymal stem cells and the generation of connective tissue cells. In fact, *Zip7-*deficiency in human mesenchymal stem cells leads to ER stress, resulting in defects in fibrogenic and chondrogenic lineage differentiation [37]. However, although *Zip13*-deficient mice show defective formation of connective tissue [38], the effect of mesenchymal stem cells on adipocyte differentiation has not been investigated. Recent research demonstrated that the inguinal white adipose tissue (iWAT) in *Zip13*-knockout mice shows a browning phenotype that reflects increased energy expenditure, thereby reducing iWAT mass. Further analysis revealed that *Zip13*-deficiency causes stabilization of C/EBP-β, an essential transcription factor for adipocyte browning, and thus promotes this process. These data suggest that the ZIP13-zinc axis plays a specific role in clearing C/EBP-β proteins to inhibit adipocyte browning [82]. 

## 4. Conclusions

Skin plays an important role as a primary defense line to protect the human body from the external environment. Healthy skin is an important factor that affects social activities and quality of life. It has long been known that zinc is important for maintaining healthy skin. Studies using knockout mouse models have demonstrated that specific zinc signaling axes mediated by individual zinc transporters play crucial roles in skin homeostasis and development. Aging is associated with reduced zinc levels in the human body. Therefore, the development of technology to control the versatile functions of zinc transporters in relation to anti-aging will contribute to regeneration of aged skin.

## Abbreviations

AE	acrodermatitis enteropathica
ATP	adenosine triphosphate
EDSSPD3	Ehlers–Danlos syndrome spondylodysplastic type 3
iWAT	inguinal white adipose tissue
MTs	metallothioneins
ZIPs	Zrt- and Irt-like proteins
ZnTs	zinc transporters
